# Billing and insurance-related administrative costs in United States’ health care: synthesis of micro-costing evidence

**DOI:** 10.1186/s12913-014-0556-7

**Published:** 2014-11-13

**Authors:** Aliya Jiwani, David Himmelstein, Steffie Woolhandler, James G Kahn

**Affiliations:** Super Models for Global Health, Arlington, VA USA; City University School of Public Health at Hunter College, New York, NY USA; Philip R. Lee Institute for Health Policy Studies, University of California San Francisco, San Francisco, CA USA; Super Models for Global Health, Oakland, CA USA

**Keywords:** Administrative costs, Healthcare financing, Health insurance

## Abstract

**Background:**

The United States’ multiple-payer health care system requires substantial effort and costs for administration, with billing and insurance-related (BIR) activities comprising a large but incompletely characterized proportion. A number of studies have quantified BIR costs for specific health care sectors, using micro-costing techniques. However, variation in the types of payers, providers, and BIR activities across studies complicates estimation of system-wide costs. Using a consistent and comprehensive definition of BIR (including both public and private payers, all providers, and all types of BIR activities), we synthesized and updated available micro-costing evidence in order to estimate total and added BIR costs for the U.S. health care system in 2012.

**Methods:**

We reviewed BIR micro-costing studies across healthcare sectors. For physician practices, hospitals, and insurers, we estimated the % BIR using existing research and publicly reported data, re-calculated to a standard and comprehensive definition of BIR where necessary. We found no data on % BIR in other health services or supplies settings, so extrapolated from known sectors. We calculated total BIR costs in each sector as the product of 2012 U.S. national health expenditures and the percentage of revenue used for BIR. We estimated “added” BIR costs by comparing total BIR costs in each sector to those observed in existing, simplified financing systems (Canada’s single payer system for providers, and U.S. Medicare for insurers). Due to uncertainty in inputs, we performed sensitivity analyses.

**Results:**

BIR costs in the U.S. health care system totaled approximately $471 ($330 – $597) billion in 2012. This includes $70 ($54 – $76) billion in physician practices, $74 ($58 – $94) billion in hospitals, an estimated $94 ($47 – $141) billion in settings providing other health services and supplies, $198 ($154 – $233) billion in private insurers, and $35 ($17 – $52) billion in public insurers. Compared to simplified financing, $375 ($254 – $507) billion, or 80%, represents the added BIR costs of the current multi-payer system.

**Conclusions:**

A simplified financing system in the U.S. could result in cost savings exceeding $350 billion annually, nearly 15% of health care spending.

**Electronic supplementary material:**

The online version of this article (doi:10.1186/s12913-014-0556-7) contains supplementary material, which is available to authorized users.

## Background

In a well-functioning health care system, sound administration is required to ensure efficient operations and quality outcomes. In the United States however, the complex structure of health care financing has led to a large and growing administrative burden [1]. In 1993, administrative personnel accounted for 27% of the health care workforce, a 40% increase over 1968 [2]. Similarly, administrative costs as a percentage of total health care spending more than doubled between 1980 and 2010 [3]. Private insurers’ overhead costs have also increased sharply, rising 117 percent between 2001 to 2010 [4].

In the U.S. multi-payer system, insurers’ coverage, billing and eligibility requirements often vary greatly, requiring providers to incur added administrative effort and cost [5]. These payment-related activities can be termed “billing and insurance-related” (BIR) [6]. On the provider side, BIR activities include functions related to interacting with payers, including filing claims, obtaining prior authorizations, and managed care administration. On the payer side, most administrative functions are billing related, with only a small portion spent on care-related issues [7]. Insurers’ profits also contribute to BIR costs.

Several studies have used micro-costing methods — cost estimates constructed from detailed classification of resource use or expenditures — to quantify the portion of administrative costs attributable to BIR activities in physician and hospital sectors. Though the specific set of methods used to estimate this cost varies by study, the general approach has been to identify the administrative functions related to BIR activities and use clinician interviews and/or surveys to determine the proportion of work time spent on these activities. In some studies, this process has been supplemented with additional interviews with non-clinical staff [8,9] and observations of work flows [9]. In California in 2001, the BIR component of administrative costs was as high as 61% for physicians (constituting 14% of revenue) and 51% for hospitals (6.6-10.8% of revenue) [7], with predominantly non-BIR activities such as scheduling and medical records management forming the rest of administrative spending. When adjusted to a standard definition of BIR, two other studies attributed 10-13% of revenue in physicians’ offices to BIR costs [9,10].

Though studies have documented BIR costs in physician and hospital sectors, the specific analytical methods and components included in the analyses vary, rendering estimates mostly non-comparable. Thus, results cannot be easily combined into a system-wide estimate. To address this problem, we synthesized available micro-costing data on BIR costs. We use an explicit, consistent, and comprehensive definition of BIR to calculate BIR costs in well-studied sectors; estimate the portion of BIR spending in other provider sectors; and present a system-wide estimate of total BIR costs in the U.S. health care system in 2012. We also calculate potential savings from a system with simplified financing, by comparing measured BIR in US health care sectors to lower levels observed with different financing mechanisms. This paper updates preliminary information developed for an Institute of Medicine roundtable on Value and Science-Driven Health Care [6]. It is intended to facilitate policy discussions about reducing the BIR component of administrative costs.

## Methods

### Overview

Drawing on U.S. National Health Expenditures (NHE), existing research and publicly reported data, we estimated total and added BIR costs in the U.S. health care system in 2012. Our estimates included the following sectors: physician practices, hospitals, private insurers, public insurers and “other health services and supplies.” We assembled micro-costing estimates of total and added BIR costs from various studies [5,8], as well as the percentage of revenue spent on BIR [5,7,9,10]. We reconciled differences in methods and findings by adjusting estimates to include the same BIR activities, payers and cost categories (detailed below). We calculated total BIR costs for each sector as the product of the 2012 U.S. NHE for that sector and the proportion of that sector’s revenue used for BIR. To calculate added BIR costs, we adjusted our total estimates using benchmarks from simplified financing systems (detailed below). To assess the effect of input uncertainty, we performed multiple sensitivity analyses.

### Health system sectors

We defined the sectors using categories designated as “personal health care” in the Centers for Medicare and Medicaid Services’ (CMS) accounting of NHEs, and in the case of payers, from the categories designated as “health insurance” [11] (Additional file 1: Table S1). Examples of categories included under “other health services and supplies” sector were nursing care, home health care, prescription drugs, and other medical products.

### Total BIR

We calculated total BIR costs for each sector as:

$$ \mathrm{Total}\ \mathrm{B}\mathrm{I}\mathrm{R}\ \mathrm{costs} = 2012\ \mathrm{N}\mathrm{H}\mathrm{E} \times \%\ \mathrm{r}\mathrm{evenue}\ \mathrm{f}\mathrm{o}\mathrm{r}\ \mathrm{B}\mathrm{I}\mathrm{R} $$

For example, 2012 projected NHE for physician and clinical services was $542.9 billion [11] and estimated average BIR costs for physicians as a percent of their gross revenues was 13% [5,7,9]. Thus, we calculated total BIR costs for physician practices in 2012 as $70.6 billion.

Existing micro-costing estimates of BIR costs in physician practices vary substantially due to differences in analytic methods and BIR functional areas included in the analyses [5,7-9,12]. Rather than select only the estimates that were obtained based on the same BIR definition and analytic method, we undertook a systematic process to make evidence more directly comparable. To do this, we classified BIR into sub-components by type of cost (e.g. contracting, insurance verification, service coding, billing, information technology, overhead) and payer (e.g., private, public). We adjusted each cost study as necessary to include all costs (e.g., overhead) and payers (e.g. public payers), based on data from other cost studies and from the NHE. For example, our estimate of 13% revenue for BIR costs for physician practices is based on a synthesis of three published studies [5,7,9]. It includes BIR costs at multi-specialty, single-specialty primary care, and single-specialty surgical practices. Each study was adjusted for missing information. In the study by Morra and colleagues, the reported estimate of BIR costs at 8.5% of revenue accounted for both public and private payers, but did not include the full range of BIR functional areas in physician practices [5]. Thus, we adjusted the Morra estimate to include information technology, time for insurance verification, a portion of clinician coding of services, and overhead attributed to BIR administration. This translated to a total BIR of approximately 13.3% of revenue, or 12.2% of revenue if clinician coding is omitted. See Additional file 1: Table S2, for details of the synthesis transformations.

We estimated that 8.5% of hospital revenue goes towards BIR activities, based on the mid-point value for hospitals found by Kahn and colleagues [7]. For public insurers, we estimated 3.1% of revenue for BIR, which is the blended mean overhead for Medicare and Medicaid [13]. Since the majority of administrative functions for private insurers are BIR, we assumed the full value of private insurer overhead, including profits, as the percentage of revenue for BIR. We estimated this at 18%, which we calculated as the total enrollment-weighted mean overhead for the 19 largest for-profit, publicly-traded insurers based on market capitalization [14], using 2010 data filed with the Securities Exchange Commission (SEC). Our estimate of private insurer BIR costs includes the administrative costs of private insurers for their administration of Medicare Advantage, Medicare Part D and Medicaid managed care. We added these costs from the 2011 historical NHE to the total estimate of BIR for private insurers.

Recent data on BIR costs for categories within the “other health services and supplies” sector is absent from the literature, though some earlier data on total administrative costs is available. An analysis of 1999 data from a sample of nursing homes in California and home health agencies across the U.S. found administrative expenditures of approximately 19% and 35% of total expenditures, respectively [15]. We conservatively assumed that 10% of revenue for our other health services and supplies sector categories goes to BIR activities, which is the mean percentage from physician practices and hospitals. We vary these assumptions in sensitivity analyses.

Table 1 shows the NHE and percent of revenue attributed to BIR for each sector.

**Table 1:** **2012 U.S. National Health Expenditures, percent billing and insurance-related (BIR), and BIR proportion considered “added”**

**Sector**	**2012 NHE (projected, in billions)**	**% for BIR costs**	**Source/reference for % BIR**	**Proportion considered “added”**	**Source for proportion added**
Physician practices	$542.9	13%	Three studies of BIR costs in physician practices in the U.S. [5,7,9]	0.73	U.S. & Canadian physician survey [5]
Hospitals	$873.1	8.5%	Midpoint BIR for hospitals [7]	0.73	U.S. & Canadian physician survey [5]
Other health services and supplies	$938.8	10%	Assumption (mean percentage from physician practices and hospitals)	0.73	U.S. & Canadian physician survey [5]
Private insurers	$884.4	18%	2010 mean admin expenses, including profit, as % premium revenue for a sample of large and small private insurers, weighted by insured enrollment (Authors’ analysis of SEC data)	0.92	Private insurer overhead vs. Medicare overhead (1.5%) [13]
Public insurers	$1,139.5	3.1%	Blended average overhead for Medicare (1.5%) and Medicaid (4.6%) [13] based on authors’ calculations using 2011 NHEA data	0.52	Public insurer overhead vs. Medicare overhead [13]

### Added BIR

We defined added BIR as the costs of BIR activities that exceed those in systems with simplified BIR requirements. For physicians, hospitals and other providers, we used Canada’s single-payer system for comparison. For private and public insurers, we used U.S. Medicare as a comparator.

We calculated added BIR costs for physicians, hospitals and other health services/supplies as:

$$ \begin{array}{c}\hfill Added\ BIR = Total\ BIR\  in\ U.S.\  sector \times \hfill \\ {}\hfill \left(\mathrm{B}\mathrm{I}\mathrm{R}\ \mathrm{in}\ \mathrm{U}.\mathrm{S}.\ \mathrm{sector}\ \hbox{--}\ \mathrm{B}\mathrm{I}\mathrm{R}\ \mathrm{in}\ \mathrm{Canadian}\ \mathrm{sector}\right)/BIR\  in\ U.S.\  sector\hfill \end{array} $$

Morra and colleagues estimated annual BIR costs in physicians’ practices at $82,975 per physician in the U.S. versus $22,205 in Ontario, Canada [5], i.e., 73% lower. While data on BIR costs in U.S. hospitals exists [7], we found no comparable data on Canadian hospitals or Canadian or U.S. non-physician health service or supply sectors. We assumed an added proportion of 73% for these sectors. We varied these assumptions in sensitivity analyses.

For private and public insurers, we calculated added BIR costs as:

$$ \begin{array}{c}\hfill Added\  insurer\ BIR = Total\  insurer\ BIR \times \hfill \\ {}\hfill \left(\mathrm{Insurer}\ \mathrm{overhead}\ \hbox{--}\ \mathrm{U}.\mathrm{S}.\ \mathrm{Medicare}\ \mathrm{overhead}\right)/ Insurer\  overhead\hfill \end{array} $$

Table 1 summarizes the proportion considered as the added BIR costs of the U.S. multi-payer system for each health care sector.

### Sensitivity analyses

#### Excluding clinician coding of services

BIR obligations likely require additional coding by clinicians, beyond that needed for clinical documentation, consuming up to 2.3% of physician revenue [9]. In our base case estimate of BIR costs in physician practices, we included 50% of the cost of coding. If we exclude clinician coding of services as a BIR function, we calculate a revised estimate of 12% for the percentage of physician revenue spent on BIR, based on the average of three studies [5,7,9].

#### Canadian medicare

In the base case analysis, we use U.S. Medicare as a comparison system against which to estimate the added BIR costs of private and public insurers. Due to differing estimates of U.S. Medicare overhead (i.e. excluding versus including private insurer administration of medical plans) [16], we explored the effect of using Canada’s Medicare as an alternative comparator to calculate the excess BIR costs of U.S. insurers. We used an overhead estimate for Canada’s Medicare of 1.8% (2011 forecast) [17] (Additional file 1: Table S3).

#### Total BIR

Due to uncertainty in some sector-specific inputs, we varied the percentage of revenue for BIR for each sector to obtain a plausible range of total BIR costs. Where available, we used lower and upper bound estimates from the literature; where unavailable, we varied the estimates by up to ten percentage points, using wider variations when data was least certain, e.g., for the “other health services and supplies” sector. Varying the estimates in tandem, we obtained upper and lower bound estimates of total BIR costs across the U.S. health care system in 2012 (Additional file 1: Table S4).

#### Added BIR

For the “other health service and supplies” sector, we varied our baseline estimate of 27% (Canadian: U.S. BIR costs) by 5 percentage points in either direction. For the hospital sector, we calculated a new ratio of 8.1% (Canadian: U.S. BIR costs) based on published data on total (not just BIR) hospital administrative spending in the U.S. and Canada [15] (text on added costs, Additional file 1).

### Ethics statement

This research did not involve human subjects and thus did not require ethics committee review.

## Results

### Total BIR

**Figure 1:**
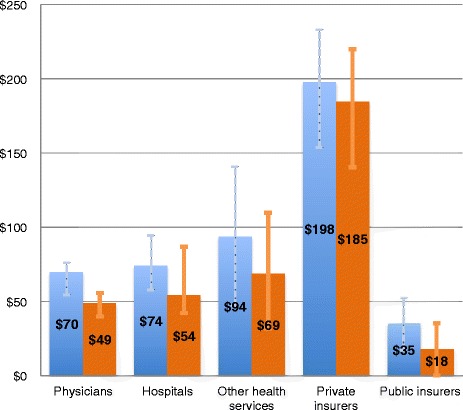
**Total and added BIR costs (billions) by health care sector.** Blue = total BIR; Orange = added BIR. Added defined as spending above indicated benchmark comparison. Physicians: synthesis range = $68-71 billion (total), $45-52 billion (added). Private insurer total and added BIR includes administrative costs incurred for privatized Medicare and Medicaid services ($24.5 billion and $14.2 billion, respectively).

Our base case calculation is that BIR costs in the U.S. totaled $471 billion in 2012. Physicians’ practices spent $70 billion on BIR activities, hospitals spent $74 billion, and the “other health service and supplies” sector spent an estimated $94 billion (Figure 1). Private insurers contributed the largest share of BIR costs, $198 billion; public insurers contributed $35 billion.

### Added BIR

About $375 billion (80%) of annual BIR costs constitutes additional spending compared to a simplified financing system. This 80% reflects 73% savings among provider sectors [5] and 93% savings in the private insurance sector. When compared to Canada’s single payer system, added BIR costs in U.S. physicians’ practices totaled $49 billion annually (Figure 1; Additional file 1: Table S3). In U.S. hospitals and “other health service and supplies” sectors, added BIR costs were $54 billion and $69 billion, respectively. When compared to BIR costs in U.S. Medicare, additional annual spending on BIR for private and public insurers totaled $185 billion and $18 billion, respectively (Figure 1).

**Figure 2:**
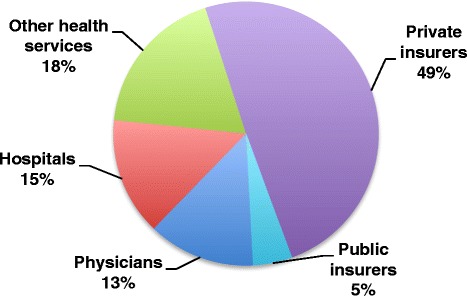
**Percentage of total U.S. added BIR costs by health care sector.** Percentages indicate contribution towards total added BIR in the U.S. ($375 billion). Added is defined as spending above indicated benchmark comparison (Canada’s single payer system for physicians, hospitals and other health services and supplies; U.S. Medicare for public and private insurers).

Figure 2 shows each health care sector’s share of total added BIR costs. Private insurers contributed much of added BIR spending at 49%, though providers collectively represented nearly half of the total.

### Sensitivity analysis

#### Excluding clinician coding

If clinician coding of services is excluded as a BIR activity, total and added BIR costs in physicians’ practices are reduced minimally to $65 billion and $48 billion, respectively.

#### Canadian medicare

Using Canada’s Medicare overhead (1.8%) [17] instead of U.S. Medicare’s (1.5%) [13] for comparison reduces added BIR costs for private and public insurers to $182 billion and $15 billion, respectively, yielding a revised estimate of $369 billion in overall added BIR costs (Additional file 1: Table S3).

#### Total BIR

Varying in tandem each of the sector-specific estimates of the percentage of revenue spent for BIR yields a plausible range for total 2012 BIR costs of $330 billion - $597 billion (Additional file 1: Table S4).

#### Added BIR

Varying the BIR cost ratios for the hospital and non-physician health service and supplies sectors as described above, and using the lower and upper bound estimates of total BIR in each sector, we obtained a plausible range for overall added BIR costs in the U.S. of $254 - $507 billion in 2012 (see Table 2).

**Table 2:** **Lower and upper bound estimates of added billing and insurance-related (BIR) costs in the U.S. health care system** ^**a**^

**Sector**	**Lower bound of added BIR** ^**b**^	**Upper bound of added BIR** ^**c**^	**Basis for added BIR ratio**
Physicians	$40 billion	$56 billion	Assumes base case ratio of BIR costs for physicians of 27% (Canada:U.S.) [5]. Lower and upper bounds assume, respectively, 10% [9] and 14% [7] estimates of % revenue BIR for physicians from published data.
Hospitals	$42 billion	$87 billion	Base case and lower bound assumes ratio of BIR costs of 27% (Canada:U.S.) [5]; upper bound assumes 8.1% (author calculation using data from Woolhandler et al, 2003 [15]).
Other health services and supplies	$32 billion	$110 billion	Base case assumes ratio of BIR costs of 27% (Canada:U.S.); lower bound assumes 32%, upper bound 22%.
Private insurers	$140 billion	$220 billion	Lower bound assumes overhead % ratio =1.5 [13]/13 (Medicare: minimum private insurer est.); upper bound assumes ratio =1.5 [13] /22 (Medicare: maximum private insurer est.). Private insurer portion of Medicare and Medicaid admin costs added directly to totals for added BIR.
Public insurers	$0 billion	$35 billion	Lower bound est. assumes an overhead % ratio =1.5/1.5 (Medicare: Public insurers); upper bound assumes overhead % ratio =1.5/4.6.
**TOTAL**	**$254**	**$507**	

^a^Added BIR costs defined as the costs of BIR activities that exceed those in a system with simplified BIR requirements (Canada’s single payer system for physicians, hospitals and “other health services and supplies”; U.S. Medicare for public and private insurers).

^b^Calculated using lower bound estimates of *total* BIR in the setting of interest; See Additional file 1.

^c^Calculated using upper bound estimates of *total* BIR in the setting of interest; See Additional file 1.

## Discussion

While published data exist on BIR costs for certain health care sectors, these isolated estimates do not provide the comprehensive portrayal needed to understand the overall costs of BIR in the U.S. health care system. First, studies of similar sectors have examined a varying set of BIR activities and costs, complicating straightforward comparisons and simple aggregation of existing component BIR costs. Akin to the tale of the blind men and the elephant [18], knowing bits of information about a few isolated pieces cannot construct an accurate picture of the whole. Second, and equally important, evidence on BIR costs in provider sectors other than physicians’ practices and hospitals is lacking from the published literature. Taken together, these realities have made it difficult for policymakers to grasp the total magnitude of health care administrative costs due to BIR activities. Our analyses, which synthesize available micro-costing data on BIR costs using a consistent definition of BIR and extrapolate data to sectors lacking estimates, present the first system-wide estimate of total BIR costs across the U.S. health care system.

Synthesizing data from existing studies, our analyses indicate that BIR costs totaled $471 billion annually in the U.S in 2012; 80% of this represents additional costs when compared to a simplified financing system. If BIR costs were pared to that of benchmark systems, system-wide savings would exceed $350 billion per year.

**Figure 3:**
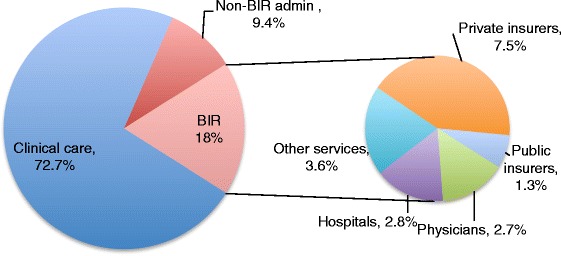
**Allocation of spending for clinical care and administration in the U.S. health care system.** Values represent share of 2012 U.S. Health Consumption Expenditures (minus government public health activities; i.e., ~$2.6 trillion). BIR = billing and insurance-related costs. Non-BIR admin = all other administrative costs, e.g., medical records, scheduling. Non-BIR admin estimate detailed in Additional file 1.

Total BIR costs currently represent about 18% of U.S. health care expenditures (excluding government public health activities). Non-BIR administrative activities represent an additional 9.4% [7], leaving less than 73% of spending for clinical care (Figure 3; details of estimation of non-BIR administrative costs in Additional file 1). Added BIR costs of $375 billion translate to 14.7% of U.S. health care expenditures in 2012, or 2.4% of GDP [19].

Our findings update and expand on previous estimates. Woolhandler et al. estimated total administrative spending in the U.S. health care system in 1999 at $294.3 billion, with added spending of $209 billion when compared to Canada [15]. Adjusting their estimates to 2012 health spending yields estimated added costs of approximately $448 billion, an estimate that falls within the upper bound of our sensitivity analysis. The earlier study assessed total administrative costs, not just BIR spending, and hence is not directly comparable to this study. However, a simplified payment system that blunts entrepreneurial incentives (as in Canada) might also reduce non-BIR administrative costs for such items as marketing and internal cost accounting.

Our estimates of BIR costs in physicians’ practices are higher than previous studies, due to the more complete set of BIR activities, payers, and costs quantified in our analysis. Morra et al. estimated total BIR costs per U.S. physician of $82,975, translating to total and added BIR costs of $38 billion and $28 billion, respectively [5]. However, their analysis involved just a subset of BIR activities, as detailed above. Similarly, Heffernan et al’s [8,12] analysis, which was limited to private payers, estimated added BIR costs of $26 billion. After adjustment to encompass the entire scope of BIR activities, payers, and overhead costs, these earlier estimates are consistent with ours (Methods and Additional file 1: Table S2). Remaining differences are small – less than 5% of total BIR costs – and most likely explained by nuances in the questions used to obtain BIR costs. We present synthesis mid-point estimates of this small variation for all relevant analyses (Additional file 1: Table S2).

Several caveats apply to our estimates. First, BIR estimates in the published literature are most robust for physicians’ practices, with limited information available for hospitals and almost no data for categories within the sector defined as “other health service and supplies.” Hence, we explored the effect of uncertainty in our estimates in sensitivity analyses.

Second, our analyses assume that BIR can be distinguished from other administrative functions. This seems a fair assumption, given that consistent findings were obtained for similar activities using varied methods. Qualitative claims by physicians of the burden of BIR lend further support to this assumption [10].

Finally, our estimate of total and added BIR is likely conservative on three accounts. First, we assume no BIR spending outside of the direct health sector, e.g., by employers or patients. Since employment-based coverage is pervasive in the U.S., documentation of BIR costs of employers might well augment the estimates presented here. Second, for providers, we assume that costs such as public relations and marketing are incurred for non-BIR reasons. This assumption might underestimate added BIR costs, since such non-BIR administrative costs might also be lower in a simplified financing system. Finally, new evidence comparing total hospital administrative costs in the U.S. to Canada and other OECD countries suggests that added BIR for hospitals may be substantially higher than our base case estimate [20].

Since these added costs are a function of the structure of the U.S. multi-payer system, some might characterize these costs as excess, in that that they provide little to no added value to the health care system. If BIR functions produce secondary benefits, such as enhanced quality or utilization management, the high BIR costs in the U.S. might be justified. Some research suggests, for example, that prior authorization can reduce over-utilization of brand-name medications without reducing patient satisfaction [21]. It is also possible that BIR functions provide benefits that have not yet been quantified. Nonetheless, any unmeasured benefit would have to be large to offset added BIR costs. Moreover, at least one study has found that higher administrative costs are associated with lower quality [22]. Hence, reducing BIR costs by adopting a simplified financing system would provide substantial recurring savings and produce an unequivocal benefit from a societal perspective. It is worth noting also that a simplified financing system does not preclude utilization controls, and that such controls might be employed in single payer systems while maintaining lower BIR costs.

Eliminating added BIR costs of $375 billion per year (14.7% of US health care spending) would provide resources to extend and improve insurance coverage, within current expenditure levels. Since uninsured individuals have utilization of about 50% of insured individuals [23], the current 15% uninsured could be covered with roughly half of the $375 billion. Remaining savings could be applied to improved coverage for those already insured. Full financial analyses of single payer insurance reform formalize and extend these analyses [24].

Unfortunately, recent reforms incorporated in the Affordable Care Act (ACA) and the American Recovery and Reinvestment Act (ARRA) are unlikely to substantially reduce BIR costs and administrative burden. Data on the BIR portion of administrative costs is not yet available in the published literature. Using the BIR cost percentages identified in this analysis as a starting point, we projected BIR costs under the ACA in 2014 and 2018. Our projections were based on estimated increases in the insured population in each health sector (i.e., 7 million more people covered by private insurance and 8 million more by Medicaid in 2014; 13 and 12 million more, respectively, in 2018) [25]. Assuming parallel increases in healthcare utilization, stable administrative complexity, and an initial cost of $5.8 million to operate the exchanges [26,27], we estimate that implementing the ACA will increase system-wide BIR costs by 5 − 7% ($24 − $34 billion) in 2014 and 9 − 11% ($45 − $55 billion) in 2018 (in 2014 USD).

Moreover, greater use of deductibles under the ACA will likely further increase administrative costs, since each claim will require processing and value adjustment before determining whether the deductible has been met. Thus, the new system will incur some new BIR costs for both the insured and uninsured portion of care. Empirical evidence from similar reform in Massachusetts is not encouraging: exchanges added 4% to health plan costs [28], and the reform sharply increased administrative staffing compared with other states [29].

While it was hoped that the ARRA’s incentives for adoption of health information technology (HIT) would reduce costs, partly by streamlining billing and administration [30], savings have not materialized [31,32]. Indeed, it appears that HIT will impose hefty implementation and training costs [33], and may require ongoing expenditures for IT upgrades and maintenance [4]. Moreover, the ACA’s emphasis on financial incentives such as pay-for-performance may well increase administrative complexity, and hence costs [34].

A recent estimate suggests that simplifying administrative activities within the existing multi-payer system by implementing a range of standardization, automation and enrollment stabilization reforms could save $40 billion annually [35]. While these savings are significant, we estimate that the annual administrative savings under a single-payer system would be nearly nine-fold higher. Though some argue that shifting to a single payer system could propagate unintended financial hazards (i.e., overutilization) and inefficiencies, as discussed previously, utilization controls can be employed in simplified financing systems while also keeping BIR costs down. Moreover, evidence from the U.S. Medicare program and the systems of several other countries [1] demonstrates that large, unified payers can achieve significantly greater efficiencies than multi-payer systems. Unified payment schemes enjoy economies of scale, sharply reduce the burdens of claims processing, and obviate the need for marketing, advertising and underwriting expenses.

## Conclusions

While the estimates presented here should continue to be refined through additional sector-specific research on BIR costs, the cost burden of BIR activities in the existing U.S. multi-payer health care system is clear. Implementation of a simplified financing system offers the potential for substantial administrative savings, on the order of $375 billion annually, which could cover all of the uninsured [36] and upgrade coverage for the tens of millions who are under-insured. Further research into the costs of BIR activities to employers and in areas such as home health care, nursing home care, and prescription drugs would augment the findings from this analysis. Data on BIR costs since implementation of the ACA is also needed to further illuminate the administrative effects of recent health reforms and provide additional tangible information for policy decision-making.

## Additional file

Additional file 1:
**Additional information and calculations on health care sectors and associated National Health Expenditure categories; results of data transformations of BIR in physician offices; and sensitivity analysis findings on total and added BIR.**
