# Supplementary material for: Billing and insurance-related administrative costs in United States’ health care: synthesis of micro-costing evidence
Source: BMC Health Serv Res. 2014 Nov 13;14:556. doi: 10.1186/s12913-014-0556-7 (PMC4283267; doi:10.1186/s12913-014-0556-7)
Supplement: Additional file 1: — Additional information and calculations on health care sectors and associated National Health Expenditure categories; results of data transformations of BIR in physician offices; and sensitivity analysis findings on total and added BIR. [file 12913_2014_556_MOESM1_ESM.doc]

**Additional File 1**

**Billing and insurance-related administrative costs in United States’ health care:**

**Synthesis of micro-costing evidence**

Table of Contents

Sectors and NHE categories [2](#__RefHeading___Toc256408920)

Table S1: Health care sectors and their associated National Health Expenditure categories [2](#__RefHeading___Toc256408921)

Table S2: Results of data transformations of estimates of total and added BIR in physician offices from the literature [3](#__RefHeading___Toc256408922)

Sensitivity analyses [4](#__RefHeading___Toc256408923)

Table S3: Sensitivity analysis findings based on using Canada’s Medicare as a comparison system for calculating added BIR among U.S. insurers [4](#__RefHeading___Toc256408924)

Table S4: Sensitivity analysis findings based on varying the % revenue spent on BIR; minimum and maximum total BIR (billions) [5](#__RefHeading___Toc256408925)

Allocation of spending for clinical care and administration in the U.S. health care system [6](#__RefHeading___Toc256408926)

Estimation of non-BIR administrative costs [6](#__RefHeading___Toc256408927)

References [7](#__RefHeading___Toc256408928)

# Sectors and NHE categories

## Table S1: Health care sectors and their associated National Health Expenditure categories

| **Sector (% of NHE)** | **NHE “personal health care” or “health insurance” category included** |
| --- | --- |
| **Provider** |  |
| Physician practices (19.2%) | Physician and clinical services |
| Hospitals (30.9%) | Hospital care |
| Other health services and supplies (33.2%) | Other professional services; Dental services; Other health, residential, and personal care; Home health care; Nursing care facilities and continuing care retirement communities; Prescription drugs; Other medical products (durable medical equipment and other non-durable medical products) |
| **Payer** |  |
| Private insurers (31.3%) | Private health insurance |
| Public insurers (40.4%) | Medicare, Medicaid, Other health insurance programs (Children’s Health Insurance Program, Department of Defense, and Department of Veterans’ Affairs) |

Note: Total within provider sector is less than 100%; remainder of NHE is private and public insurance overhead, government public health activities, and spending on investment (research; structures and equipment). Total within payers is less than 100%; remainder is out-of-pocket payment and spending by other third party payers (e.g., worksite health care, other private revenues, Indian Health Service, etc.)

## Table S2: Results of data transformations of estimates of total and added BIR in physician offices from the literature

|  | **Billing and insurance-related administrative costs** | | **Method** | | |
| --- | --- | --- | --- | --- | --- |
| **Study** | **Total BIR** | **Added* BIR** | **Data source(s)** | **Types of costs included** | **Basis for estimating added costs** |
| Current findings | $71 billion | $52 billion | Three studies applied to 2012 NHE. | All BIR tasks (with half of clinician service coding), all payers & costs. | Ratio based on Morra et al |
| Morra | $38 billion | $28 billion | BIR costs per physician from a U.S. representative survey, applied to 2006 data on # office-based physicians and NHE | Includes time spent on formularies, prior authorization, claims/billing, quality data and credentialing | Ratio of Canadian:US BIR spending |
| Morra (adjusted) | $68 billion | $49 billion | Adjusted % revenue towards BIR, applied to 2009 data on # office-based physicians and NHE. | Adjusted to include health IT and overhead, and time for insurance verification and clinician coding of services (50%). | Ratio of Canadian:US BIR spending |
| Heffernan | Not reported | $26 billion | Mass. General Phys. Org, applied to 2009 NHE. | All BIR tasks and costs (minus lost revenue), for private payers only; % physician revenue from private insurers from NHE 2007. | Micro-costing of current private payers vs. single set of payment rules (e.g., Medicare fee-for-service). |
| Heffernan (adjusted) | n/a | $45 billion | Mass. General Phys. Org data, applied to 2012 NHE. | All BIR tasks, adjusted to include private+public payer portion of 2012 physician NHE |  |
| Synthesis | **$68 - 71 billion** | **$45 - $52 billion** | Multiple; see above. | Similar to Kahn: all payers and BIR tasks. | Ratio based on Morra et al |

*"Added" is defined as spending above the indicated benchmark comparison.

Table S2 describes the transformations we made for two estimates from the literature that employed different analytic methods to determine BIR costs. Based on these transformations, the synthesis estimates of BIR costs in physician practices are as follows: $68-$71 billion (total BIR costs), and $45-52 billion (added BIR costs).

# Sensitivity analyses

## Table S3: Sensitivity analysis findings based on using Canada’s Medicare as a comparison system for calculating added BIR among U.S. insurers

| **Sector** | **Total BIR costs** | **Added* BIR costs** |
| --- | --- | --- |
| Physicians | $70 billion | $49 billion |
| Hospitals | $74 billion | $54 billion |
| Other health services and supplies | $94 billion | $69 billion |
| Private insurers | $198 billion | $182 billion** |
| Public insurers | $35 billion | $15 billion*** |
| **TOTAL** | **$471 billion** | **$369 billion** |

* "Added" is defined as spending above the indicated benchmark comparison.

**Based on an “added” proportion of 0.90

***Based on an “added” proportion of 0.42

## Table S4: Sensitivity analysis findings based on varying the % revenue spent on BIR; minimum and maximum total BIR (billions)

| **Sector** | **% revenue BIR (base case)** | **% revenue BIR (minimum)** | **Source** | **% revenue BIR (maximum)** | **Source** | **Total BIR costs – min (billions)** | **Total BIR costs - max (billions)** |
| --- | --- | --- | --- | --- | --- | --- | --- |
| Physician practices | 13.0% | 10.0% | Lower bound estimate from Sakowski 2009 | 14.0% | Estimate from Kahn 2005 | $54.3 | $76.0 |
| Hospitals | 8.5% | 6.6% | Lower bound estimate from Kahn 2005 | 10.8% | Upper bound estimate from Kahn 2005 | $57.6 | $94.3 |
| Other health services and supplies | 10% | 5.0% | Assumption | 15.0% | Assumption | $46.9 | $140.8 |
| Private insurers | 18% | 13.0% | Minimum 2010 admin expenses as % premium revenue from sample of 19 large insurers; authors' analysis of SEC data | 22.0% | Maximum 2010 admin expenses as % premium revenue from sample of 19 large insurers; authors' analysis of SEC data | $153.7 | $233.3 |
| Public insurers | 3.1% | 1.5% | U.S. Medicare overhead based on authors' calculation | 4.6% | U.S. Medicaid overhead based on authors’ calculation | $17.1 | $52.4 |
| **TOTAL:** |  | | | | | **$330** | **$597** |

Added BIR costs***:*** When calculating added BIR costs, published data was not available on BIR costs in Canadian hospitals. As such, we calculated a new ratio based on published data on total hospital administrative spending in the U.S. and Canada. Woolhandler and colleagues estimated total administrative spending in U.S. and Canadian hospitals at 24.3% and 12.9% of revenues, respectively ($315 vs. $103 per capita). Based on Kahn et al (2005), the non-BIR component of hospital administrative costs is 49% ; when applied to the total U.S. hospital administrative spending in Woolhandler, this translates to 11.9% of total revenue. Assuming that U.S. and Canadian hospitals spend the same percentage of total revenue on non-BIR administrative activities, we inferred that Canada spends 1% of hospital revenue on BIR costs (12.9% admin costs – 11.9% non-BIR costs), and the U.S. spends 12.4% (24.3% admin costs – 11.9% non-BIR costs). Therefore, the ratio of Canadian:U.S. BIR costs is 8.1% (=1/12.4). We used this revised estimate to examine the effect of variation in our baseline estimate of added BIR costs in hospitals.

# Allocation of spending for clinical care and administration in the U.S. health care system

## Estimation of non-BIR administrative costs

Figure 3 of the manuscript depicts the estimated allocation of spending on clinical care and administration in the U.S. health care system, assuming data on BIR costs from our analyses. We estimated non-BIR administration costs at 9.4% of 2012 national health consumption expenditures (minus government public health activities), based on published data on BIR as a percentage of total administrative costs (i.e., BIR of 61% of admin costs for physicians, 51% for hospitals, 85% for private insurers, and 83% for public insurers. In the absence of data for categories within the “other health service and supplies” sector, we assume that BIR contributes 51% of total admin costs, the same value as in hospitals). The 9.4% total non-BIR admin costs includes 1.7% for physicians, 2.7% for hospitals, 3.4% for the “other health services and supplies” sector, 1.3% for private insurers, and 0.3% for public insurers.

# References
